# The diamagnetic component map from quantitative susceptibility mapping (QSM) source separation reveals pathological alteration in Alzheimer’s disease-driven neurodegeneration

**DOI:** 10.1016/j.neuroimage.2023.120357

**Published:** 2023-09-01

**Authors:** Maruf Ahmed, Jingjia Chen, Arvin Arani, Matthew L. Senjem, Petrice M. Cogswell, Clifford R. Jack, Chunlei Liu

**Affiliations:** a Department of Electrical Engineering and Computer Sciences, University of California, Berkeley, CA 94720, USA; b Department of Radiology, Mayo Clinic, 200 First St SW, Rochester, MN 55905, USA; c Department of Information Technology, Mayo Clinic, 200 First St SW, Rochester, MN 55905, USA; d Helen Wills Neuroscience Institute, University of California, Berkeley, CA 94720, USA

**Keywords:** Alzheimer’s disease, Quantitative susceptibility mapping, Neurodegeneration, DECOMPOSE, Magnetic susceptibility, Demyelination

## Abstract

A sensitive and accurate imaging technique capable of tracking the disease progression of Alzheimer’s Disease (AD) driven amnestic dementia would be beneficial. A currently available method for pathology detection in AD with high accuracy is Positron Emission Tomography (PET) imaging, despite certain limitations such as low spatial resolution, off-targeting error, and radiation exposure. Non-invasive MRI scanning with quantitative magnetic susceptibility measurements can be used as a complementary tool. To date, quantitative susceptibility mapping (QSM) has widely been used in tracking deep gray matter iron accumulation in AD. The present work proposes that by compartmentalizing quantitative susceptibility into paramagnetic and diamagnetic components, more holistic information about AD pathogenesis can be acquired. Particularly, diamagnetic component susceptibility (DCS) can be a powerful indicator for tracking protein accumulation in the gray matter (GM), demyelination in the white matter (WM), and relevant changes in the cerebrospinal fluid (CSF). In the current work, voxel-wise group analysis of the WM and the CSF regions show significantly lower |DCS| (the absolute value of DCS) value for amnestic dementia patients compared to healthy controls. Additionally, |DCS| and τ PET standardized uptake value ratio (SUVr) were found to be associated in several GM regions typically affected by τ deposition in AD. Therefore, we propose that the separated diamagnetic susceptibility can be used to track pathological neurodegeneration in different tissue types and regions of the brain. With the initial evidence, we believe the usage of compartmentalized susceptibility demonstrates substantive potential as an MRI-based technique for tracking AD-driven neurodegeneration.

## 1. Introduction

In neurodegenerative diseases such as Alzheimer’s disease (AD), the human brain alters to a substantial extent throughout the disease’s progression. One of the familiar pathological changes in AD is the accumulation of two proteins in the brain gray matter (GM), namely, β-amyloid in the form of plaques (Lei et al., 2020; Sisodia and Price, 1995) and *τ* in the form of tangles (Pontecorvo et al., 2017). Often these plaques and tangles colocalize with iron, e.g., because of undergoing neuroinflammation (Acosta-Cabronero et al., 2018; Spotorno et al., 2020; Lee et al., 2004; Bousejra-elgarah et al., 2011; Derry et al., Jan. 2020; Telling et al., Oct. 2017). In addition, iron homeostasis may alter concurrently during the disease progression (Ndayisaba et al., 2019). In addition to these changes, alteration in the white matter (WM), which may include neurite damage, glial damage, cell death, and eventual atrophy, is known to happen in many neurodegenerative diseases such as AD (Au et al., 2021; Maier-Hein et al., 2015; Nasrabady et al., 2018), multiple sclerosis (MS) (Wisnieff et al., 2015), small vessel disease (SVD) (Pasi et al., 2016) and so on. Recently, many studies (Hansson et al., 2019; Bouwman et al., 2022) have reported the alterations of the β-amyloid content in the cerebrospinal fluid (CSF) on the course of neurodegeneration. Specifically, a decreased level of β-amyloid-42 in CSF has been reported to potentially be a prominent AD biomarker.

Positron emission tomography (PET) imaging is the gold standard to detect β-amyloid in the brain GM clinically using Pittsburg compound (PiB), and *τ*-protein using Flortaucipir (FTP) (Insel et al., 2020); however, PET imaging suffers from low spatial resolution, radiation exposure, and off-target binding (Cogswell et al., 2021).

Quantitative Susceptibility Mapping (QSM) is an MRI technique that non-invasively measures the bulk magnetic susceptibility of biological tissue. In the brain, QSM has been applied to map paramagnetic (positive susceptibility) iron distribution and alterations (Ndayisaba et al., 2019; Bulk et al., 2018; Du et al., 2018; Bulk et al., 2018; Sun et al., 2017; Möller et al., 2019). Additionally, myelin and neurodegeneration-related β-amyloid or *τ*-protein accumulations are diamagnetic and appear as negative susceptibility values in QSM (Deistung et al., 2017; Liu et al., 2015; Gong et al., 2019). Voxel-based and region-based analyses have shown statistically significant differences between AD patients and healthy control (HC) participants in deep GM such as putamen and posterior GM and WM (Spotorno et al., 2020; Kim et al., 2017); however, the QSM value of a voxel is a compounded effect of paramagnetic and diamagnetic molecular sources within a given voxel. Thus, if present within the same voxel opposing effects of iron vs. myelination or pathological protein aggregations could cancel out the QSM signal or significantly reduce it. For example, recent studies have indicated that β-amyloid and *τ*-tangles induce diamagnetic susceptibility which is the opposite of the paramagnetic susceptibility of iron (Gong et al., 2019).

Several recently proposed susceptibility separation methods (Chen et al., 2021; Shin et al., 2021; Dimov et al., 2022) have opened a new avenue to look at pathology using different susceptibility sources more effectively. In particular, the DECOMPOSE-QSM (Chen et al., 2021) postprocessing method has been developed to resolve the aforementioned sub-voxel level susceptibility mixture by calculating the paramagnetic component susceptibility (PCS) and the diamagnetic component susceptibility (DCS) using a signal model with summation of exponentials. This method uses the same MRI scan (multi-echo gradient echo) as is needed for calculating QSM, and no additional MRI contrast is required. PCS is dominantly affected by iron, and DCS can inform pathological changes caused by myelin degeneration, protein aggregation, and other species with diamagnetic susceptibility. While paramagnetic iron accumulation in the brain is one of the widely studied phenomena in healthy aging and neurodegenerative process, it is important to study its counterpart, namely the diamagnetic susceptibility or DCS alteration in AD more extensively.

In the current work, we investigate the DCS of the whole brain volume for cohorts of amnestic dementia (aDem) patients and healthy controls (HC). By using voxel-wise group comparison, we show that whole brain differences exist between aDem patients and HC participants for all GM, WM, and CSF regions. With β-amyloid and *τ* PET scans, we hypothesize that the higher voxel-wise |DCS| (the absolute value of DCS) values for aDem participants, relative to controls, in GM may result from protein aggregations; the observed lower |DCS| value in WM may be caused by demyelination and in CSF may indicate β-amyloid protein ratio change. ROI-based correlation analysis suggests demyelination may also happen in the GM because of *τ*-protein aggregation.

## 2. Materials and methods

### 2.1. Demographic data

MRI, PET, imaging data, and demographic data were collected at the Mayo Clinic Alzheimer’s Disease Research Center (MCADRC). Data from 71 β-amyloid and *τ* negative cognitively unimpaired (CU) HC participants and 49 β-amyloid and *τ* positive (Jack et al., Apr. 01, 2018) aDem patients were used in the current analysis. Relevant demographic details are presented in Table 1. HC and aDem groups were compared using the *χ*^2^ test for sex. No significant difference was found between the aDem and the HC group (*χ*^2^ = 0.64, *p* > 0.4). Hence, sex was not considered a confounding variable for any of the analyses performed in this article. The study was approved by the Mayo Clinic institutional review board. All participants provided informed written consent; a legally authorized representative provided consent for cognitively impaired participants, as necessary. MRI, PET, and other data from the MCADRC are available to qualified academic and industry researchers by request to the MCADRC Executive Committee (https://www.mayo.edu/research/centers-programs/alzheimers-disease-research-center/research-activities/mayo-clinic-study-aging/for-researchers/data-sharing-resources).

### 2.2. MRI acquisition

T_1_-weighted structural scans were performed on a 3T MRI scanner (Siemens MAGNETOM Prisma, software version: VE11C) using a 3D Magnetization Prepared Rapid Acquisition Gradient Recalled Echo (MPRAGE) (Brant-Zawadzki et al., 1992) pulse sequence. The scan parameters were: TE/TR of 3/2300 ms, TI of 900 ms, and 0.8 × 0.8 × 0.8 mm^3^ imaging resolution. The MPRAGE image for each participant was segmented using FreeSurfer 5.3 (https://surfer.nmr.mgh.harvard.edu/) (Fischl, 2012) for defining GM ROIs to be used later for correlation analysis.

3D Multi-echo GRE data (MEGRE) with 5 echoes were collected in the same exam with the following acquisition parameters: TE_1_/ΔTE/TE_5_ = 6.71/3.91/22.35 ms, matrix size = 384 × 269 × 88, spatial resolution = 0.52 × 0.52 × 1.8 mm^3^, with GRAPPA 2x acceleration; the total acquisition time per participant was 6 min 37 s. More details on the acquisition can be found in Cogswell et al. (2021).

### 2.3. Positron emission tomography (PET) acquisition

Late uptake PiB PET images for detecting β-amyloid plaques were acquired on PET-CT scanners for 40–60 minutes and Flortaucipir (FTP or *τ*) for 80–100 min after injection. Each had four, five-minute frames. Low-dose CT scanning was performed for attenuation correction. Reconstructions were performed on-scanner with iterative ordered subset expectation maximization (OSEM) algorithm. A 5 mm Gaussian post-reconstruction filter was applied, along with standard corrections for attenuation, scatter, random coincidences, and decay. Four-frame dynamic PET images were co-registered with a group-wise rigid registration to correct for cross-frame motion and averaged to produce a single static (summed) PET image. These images were co-registered with T_1_-weighted structural images using SPM12 (SPM, 2022). Voxel-wise PET SUVr values were computed by normalizing with cerebellar crus GM (Jack et al., 2017). More details on PET imaging can be found in (Cogswell et al., 2021).

### 2.4. QSM processing

QSM was calculated using STISuite (Liu, 2022). The MEGRE complex images were down sampled in-plane by a factor of two to reduce anisotropy of the spatial resolution, hence, improving the accuracy of the dipole kernel inversion for QSM and reducing computation time during the processing of DECOMPOSE-QSM. Brain Extraction Tool (BET) in FSL (Analysis Group 2022) was used to extract brain tissue from MEGRE magnitude images for all the echoes. Then Laplacian phase unwrapping (Li et al., 2014), V-SHARP background phase removal with the radius of the spherical mean value filter as 12 mm (Li et al., 2011), and STAR-QSM (Wei et al., 2015) inversion algorithms were applied to compute the final QSM maps for every echo of the MEGRE images.

### 2.5. DECOMPOSE-QSM

Details of DECOMPOSE algorithm are explained in Chen et al. (2021). In brief, the DECOMPOSE model assumes each voxel consists of paramagnetic, diamagnetic, and neutral components. The nonlinear optimization solver uses multi-echo gradient echo magnitude and QSM images to calculate the paramagnetic component susceptibility (PCS) map and the diamagnetic component susceptibility (DCS) maps. To compare DCS between groups absolute value of DCS was used to avoid complications of explanations. A higher |DCS| value means a greater amount of diamagnetic content.

### 2.6. Image registration, warping, and segmentation

Structural images were segmented using FreeSurfer (Fischl, 2022). Image registration and mask warping was performed using Advanced Normalization Tools ANTs (Avants et al., 2022). First, brain masks from the MPRAGE images were extracted using FSL BET (Smith, 2002). Then the first echo of the MEGRE magnitude images was co-registered to BET extracted MPRAGE image using rigid transformation with ANTs. These transformations were saved and FreeSurfer segmented masks were warped from structural image space to QSM image space using these transformations using ANTs for ROI analysis.

### 2.7. Voxel-wise group analysis

Structural images were warped to standard (Montreal Neurological Institute) MNI (MNI ICBM152 non-linear, 2023) space using (ANTs). For each participant, a composite transform was generated combining the transforms from QSM to MPRAGE T_1_ structural space and the one from native T_1_ structural space to MNI T_1_ space. This composite transform was used to warp an individual’s |DCS| map to MNI space, and this process was repeated for all participants. All |DCS| maps warped to MNI space were concatenated and sent as input to the FSL tool named randomize (fsl randomise, 2023) for group comparison. The disease status and age of the patients were used to generate the design matrix. Then FSL randomize was used for group comparison using the default 5000 permutations (Anderson and Robinson, 2001; randomise theory, 2023). The Threshold-Free Cluster Enhancement (TFCE) (Smith and Nichols, 2009) statistics with family-wise error (FWER) correction were reported. The randomize analysis was performed for the full brain tissue volume without spatial smoothing. MNI space was segmented using FSL FMRIB’s Automated Segmentation Tool (FAST) (Zhang et al., 2001). Region-specific results were drawn by applying tissue segmentation masks to the randomize outputs. The same process was repeated for the PCS maps too. For voxel-wise analysis, corrected *p* < 0.05 was considered.

A metric defined by the ratio of the number of voxels with a greater absolute value of corresponding susceptibility metric in aDem subjects relative to the HC participants and the number of voxels with a smaller absolute value of susceptibility for the same combination was computed. The ratio is denoted by m and subscripted by the corresponding susceptibility metric as follows:

(1) $$m_{PCS}=\frac{\text{no.}\;\text{of}\;\text{voxels}\;\text{with}\;{PCS}_{aDem}\;>\;{PCS}_{CU}}{\text{no.}\;\text{of}\;\text{voxels}\;\text{with}\;{PCS}_{aDem}\;<\;{PCS}_{CU}}$$

(2) $$m_{|DCS}=\frac{\text{no.}\;\text{of}\;\text{voxels}\;\text{with}\;\left|{DCS}_{aDem}\right|>\;\left|{DCS}_{CU}\right|}{\text{no.}\;\text{of}\;\text{voxels}\;\text{with}\;\left|{DCS}_{aDem}\right|<\;\left|{DCS}_{CU}\right|}$$

### 2.8. Region of interest (ROI) based correlation analysis

Previously, it has been shown that *τ* accumulation may be a key factor in Alzheimer’s Disease progression (Adams et al., 2019; Pontecorvo et al., 2020), hence, PCS and DCS values of regions primarily affected by *τ* pathology and altered most during the progression of the disease were used for ROI analysis in this work. ROIs were chosen based on how frequently or severely they are affected by *τ* pathology based on a review of relevant articles (Insel et al., 2020; Cogswell et al., 2021; Cho et al., 2016; Sanchez et al., 2021; Jack et al., 2018; Harrison et al., 2019). ROIs included for the analysis were the entorhinal cortex, parahippocampal cortex, amygdala, fusiform gyrus, inferior temporal cortex, middle temporal cortex, superior temporal cortex, posterior cingulate cortex, precuneus, and lingual cortex. Additionally, deep gray matter regions of the caudate nucleus, putamen, and globus pallidus were also included because of their apparent importance found in QSM literature (Du et al., 2018; Acosta-Cabronero et al., 2013; Tiepolt et al., 2020). ROI correlation analysis was performed for 48 β-amyloid and *τ* positive aDem patients only, one β-amyloid and *τ* positive aDem patient was not included due to FreeSurfer segmentation error. Rstudio (RStudio Team 2020) (Rstudio, Inc, Boston, MA) 2022.07.2 Build 576 for MacOS and R 4.2.1 were used for all ROI-based statistical analyses including the computation of linear regression and Pearson’s correlation coefficient. The p-value was corrected with considerations of multiple comparisons with 5000 permutations (Chen et al., 2013). BrainSlicer (Mascali, 2022) was used to plot brain images. For ROI analysis, corrected *p* < 0.05 (*), *p* < 0.01 (**), *p* < 0.001 (***) were considered.

## 3. Results

**Fig. 1.**
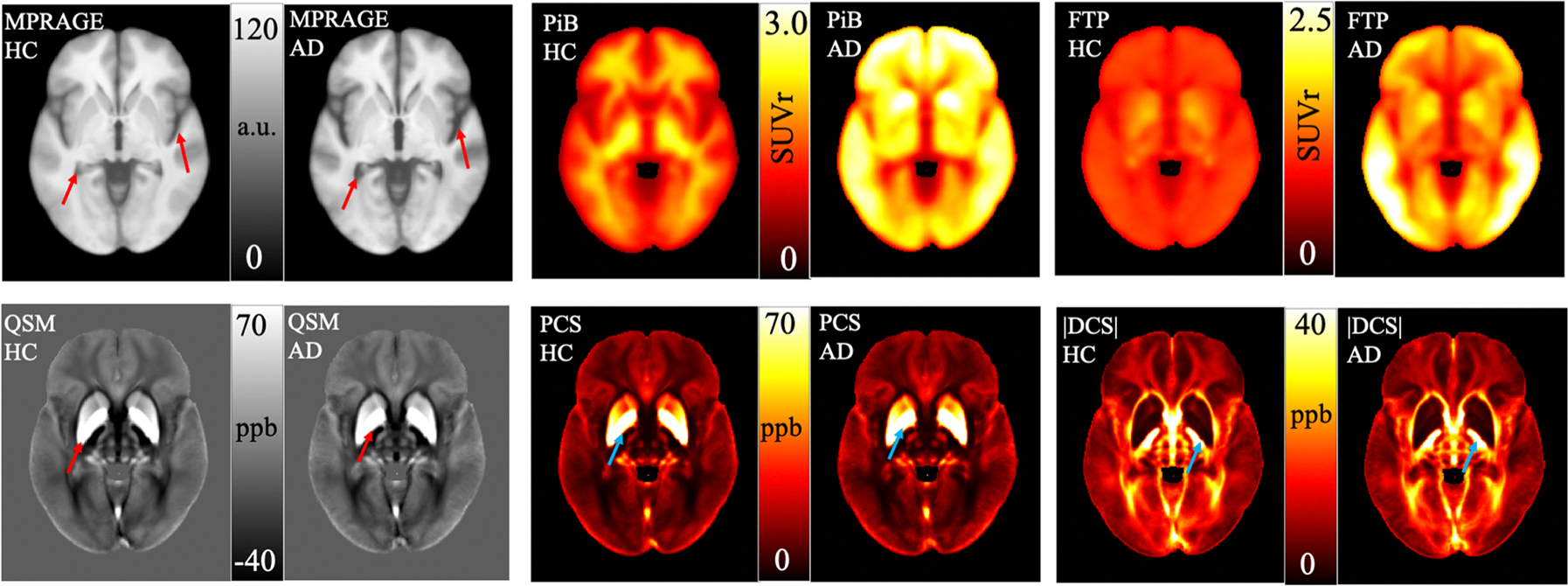
Representative slice of average images in MNI space of different contrasts for the HC and the aDem group. Ventricles appear bigger in structural images of the aDem patients compared to the HC participants as seen in the structural MPRAGE images (top left panel). Strong PiB PET (top middle) signal spread over the whole cortical GM was present for the aDem patients dissimilar to that of the HC participants which was marked by the non-specific PET SUVr signal in the WM. FTP PET SUVr (top right) was high in the temporal cortex, which is typical of the aDem patients but absent in the HC participants. QSM (bottom left) showed stronger contrast in the deep GM of the aDem patients compared to the HC participants. PCS (bottom middle) demonstrated brighter contrast in the deep GM for the aDem patients compared to the HC participants. |DCS| (bottom right) showed an overall darker contrast in the aDem group compared to the HC participants. In cortical WM, |DCS| map of the aDem group showed reduced contrast compared to the HC group. Note that the DCS map is shown in absolute value for simplicity. The higher the |DCS| value the more diamagnetic content is. a.u.: arbitrary unit. SUVr: standardized uptake value ratio. ppb: parts per billion, 10^−9^.

Fig. 1 depicts a representative axial slice of different imaging contrasts of the averaged images across subjects in the MNI space utilized in the current work. Structural MPRAGE images were used for segmentation and normalization. The averaged structural image in aDem group showed more ventricular space (red arrows) compared to HC participants in the chosen slice. PiB and FTP PET were used as pathological markers of dementia. The averaged PiB image of the aDem group showed widespread elevated SUVr in cortical GM compared to HC participants. The average FTP SUVR in aDem group was higher in temporal cortex compared to HC participants. QSM values show the bulk magnetic susceptibility relative to the reference (the mean susceptibility of the whole brain volume). A positive value (bright) means paramagnetic susceptibility and a negative value (dark) means diamagnetic susceptibility. QSM and MEGRE magnitude images were used as inputs to the DECOMPOSE-QSM algorithm. Average QSM and PCS images showed a stronger signal in basal ganglia (red and blue arrows) in aDem group compared to HC participants in the slice shown. The average |DCS| value was weaker in aDem group compared to HC participants in the internal capsule (blue arrows) region. Also, the brighter |DCS| regions had more well-defined and sharper boundaries in HC participants compared to aDem patients. Statistical comparisons are presented in the following sections. QSM, PCS, and |DCS| maps for a representative aDem patient and HC participant in their native spaces have been presented in Fig. S1. Also, standard deviation for all the modalities of images is presented in Fig. S2.

### 3.1. Voxel-wise group comparison between aDem patients and HC participants

**Fig. 2.**
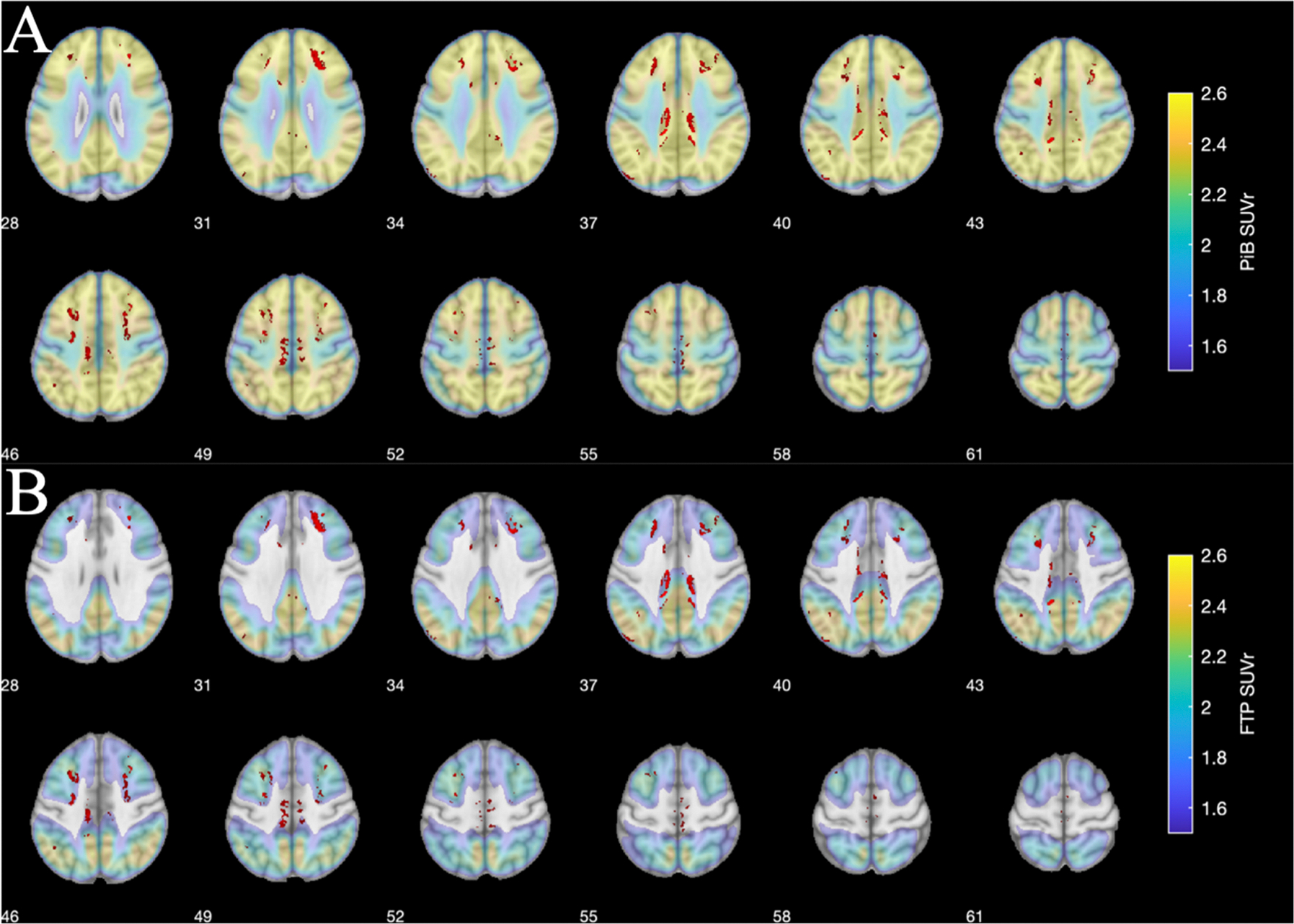
Regions of statistically significant (p<0.05) higher |DCS| value (red) in the GM of aDem patients vs CU HC participants overlaid on top of the average β-amyloid PET (A, top two rows) and average τ PET (B, bottom two rows) in MNI space. The clusters of significant differences are primarily located at superior and medial frontal gyri and paracentral gyri. Numbers in the figure denote the slice locations.

**Fig. 3.**
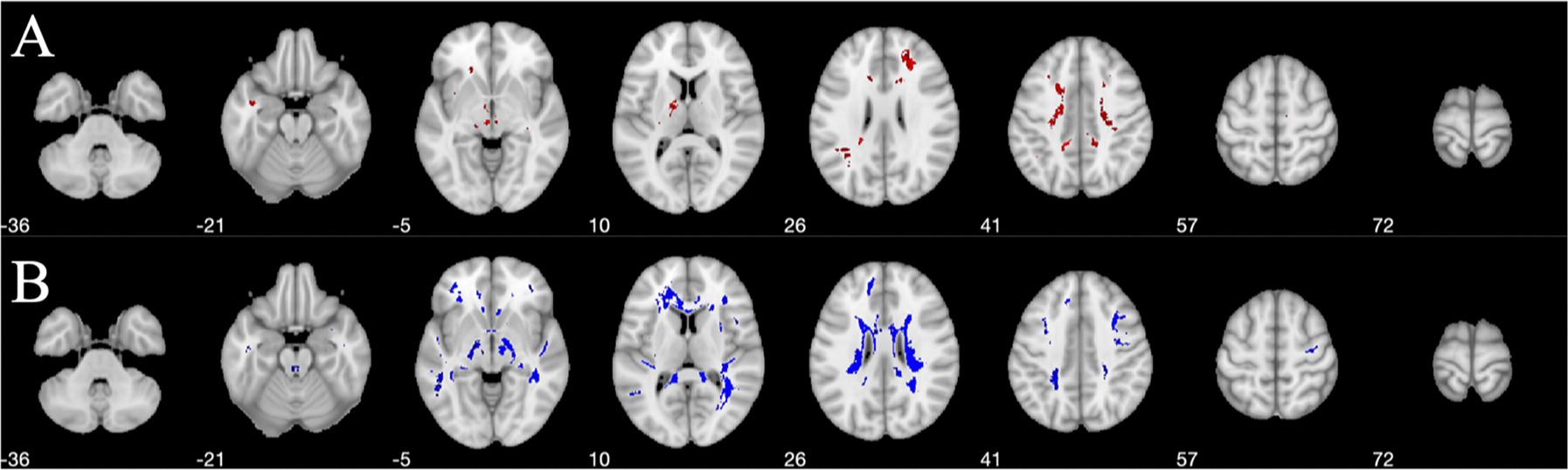
Regions of statistically significant (p<0.05) (A) higher (red) and (B) lower (blue) |DCS| value in the WM of aDem patients relative to the HC participants in MNI space. Clusters of higher |DCS| value in aDem group are primarily located in the frontal WM, CC, and IC. Clusters of lower |DCS| values are predominantly periventricular. Numbers in the figure denote the slice locations.

**Fig. 4.**
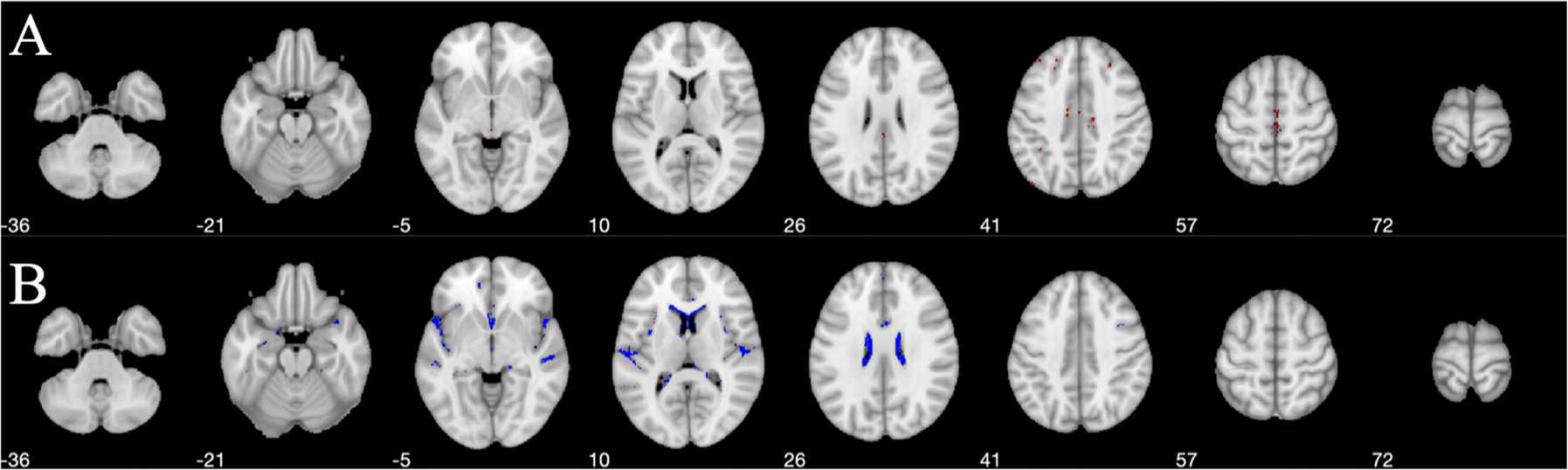
Regions of statistically significant (p<0.05) (A) higher (red) and (B) lower (blue) |DCS| value in the CSF of aDem patients compared to the HCs in MNI space. Lower |DCS| value in aDem group relative to controls primarily was observed in lateral ventricles and sylvian fissures. A similar effect was not observed in the 4th ventricle or basal cisterns. Numbers in the figure denote the slice locations.

Whole brain group comparison maps in GM, WM, and CSF revealed alterations in different regions as shown in Figs. 2, 3, and 4. |DCS| maps of β-amyloid and *τ* positive aDem patients were statistically compared to β-amyloid and *τ* negative HC participants. A higher |DCS| value means a larger amount of diamagnetic content and vice versa.

Fig. 2 shows the regions where on a group level |DCS| value was larger in aDem patients in the GM relative to the HC participants. The statistical significance cluster maps are overlaid on the top of PiB maps in Fig. 2(A) and the background is the structural MNI image. The red-colored regions show the areas where aDem patients had a statistically significant higher value of |DCS| relative to the HC participants. Similarly, in Fig. 2(B) red colored regions with statistically significant higher |DCS| values for aDem patients relative to HC participants are overlaid on top of FTP PET SUVr showing the overlap between high *τ* PET and high |DCS| values in the aDem patients. Clusters of significant difference located in the superior and medial frontal gyri overlapped with high PiB PET SUVr, whereas clusters of significant difference located in the paracentral gyri overlapped with both high PiB and FTP PET SUVr. Regions with lower |DCS| in the GM for the aDem patients relative to the HC participants have been presented in Fig. S3.

In Fig. 3, the area (number of voxels) of the WM with a lower |DCS| value was larger than the area of the WM with a higher |DCS| value in aDem patients compared to the HC participants. A lower |DCS| value in aDem patients relative to HC participants was observed primarily in the periventricular WM; a higher |DCS| value in aDem group was observed in the frontal WM, corpus callosum (CC), and internal capsule (IC).

In Fig. 4, aDem subgroup showed a lower |DCS| value in most of the CSF region relative to the HC participants, clusters residing predominantly in the lateral ventricles and sylvian fissures, but not in the 4th ventricle or basal cisterns.

Regions with a statistically significant difference in PCS for the aDem patients relative to the HC participants are presented in Fig. S4, S5, and S6 respectively for the GM, WM, and CSF.

As shown in Table 2, *m*_PCS_ was the highest in the WM, and the lowest in the GM. *m*_|DCS|_ is the lowest in the CSF and highest in the GM. The value of *m*_PCS_ being larger than unity in all the segments indicated that in aDem group paramagnetic susceptibility was greater in all the segments when compared to the HC participants. Additionally, the value of *m*_|DCS|_ being smaller than unity in all the segments alluded that in aDem group the absolute diamagnetic susceptibility was lower in all the segments when compared to the HC participants

### 3.2. Correlation between ROI susceptibility metric and τ PET

**Fig. 5.**
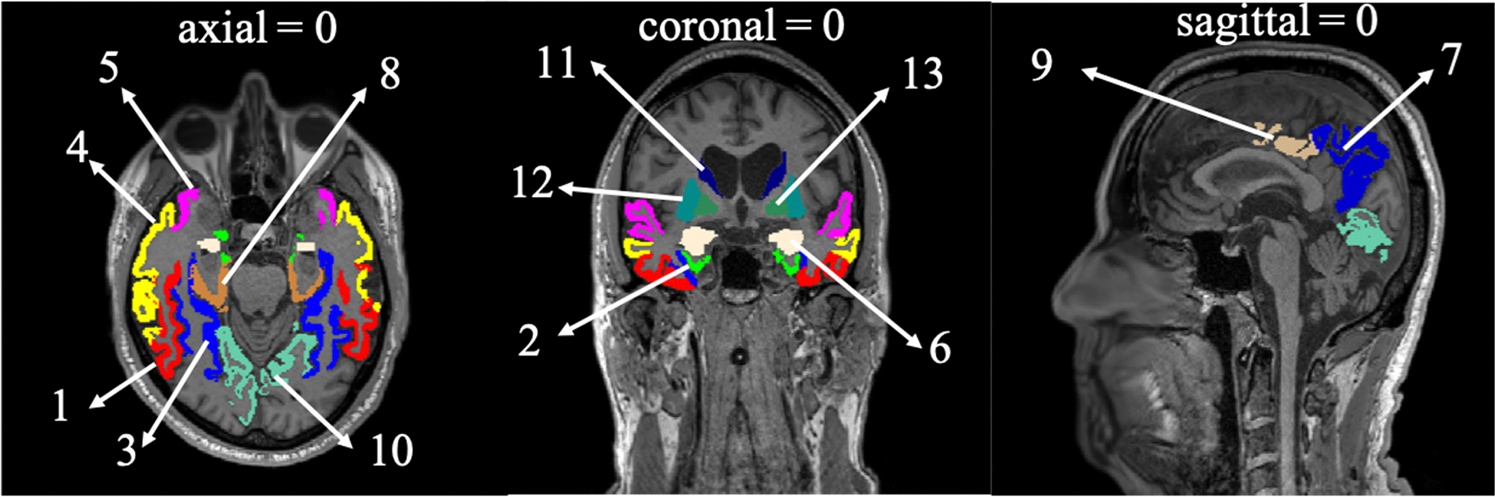
Regions included in the correlation analysis. The slice locations are shown designating the midpoint as the origin (0,0,0) of a 65 year old male amnestic dementia patient’s FreeSurfer processed structural image space with a matrix size of 256 × 256 × 256. Here, (1) Inferior temporal cortex, (2) entorhinal cortex, (3) fusiform cortex, (4) middle temporal cortex, (5) superior temporal cortex, (6) amygdala, (7) precuneus, (8) parahippocampal cortex, (9) posterior cingulate cortex, (10) lingual cortex, (11) caudate nucleus, (12) putamen, and (13) globus pallidus.

ROIs analyzed in the correlation analysis are presented in Fig. 5.

**Fig. 6.**
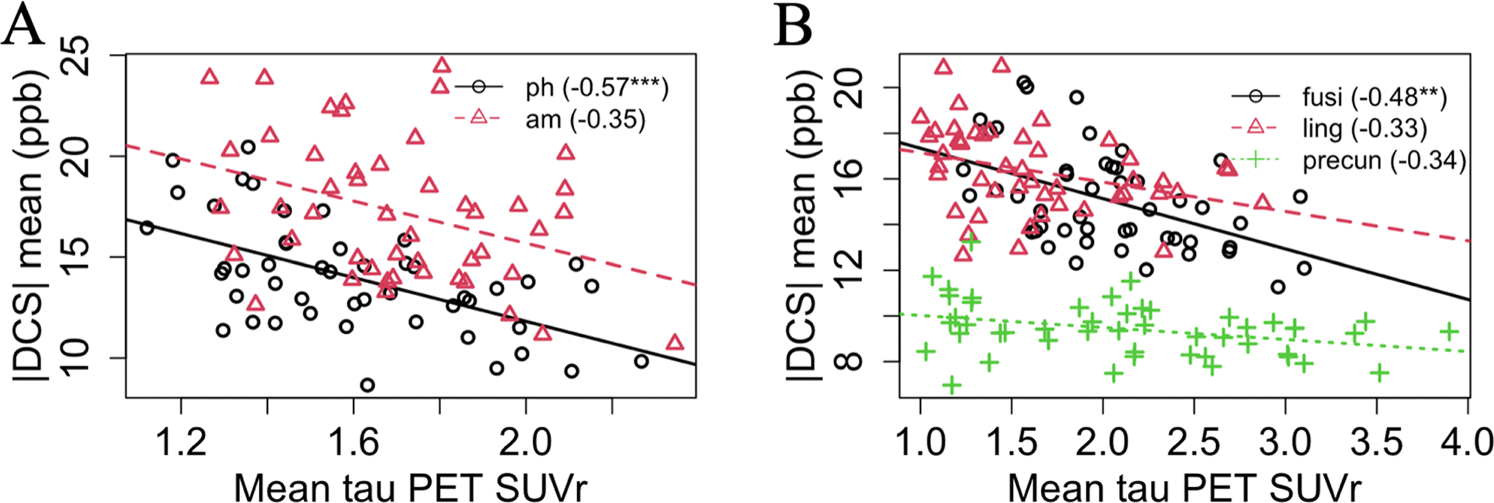
Regional association between mean |DCS| and τ PET for selected (A) limbic and (B) neocortical ROIs. The plot has been subdivided into two subplots for the sake of legibility. The number in the parenthesis represents Pearson’s correlation coefficient. Here, *p<0.05, **p<0.01, ***p<0.001, after correction for multiple comparisons using permutation tests. Also, ph = parahippocampal cortex, am = amygdala, fusi = fusiform gyrus, ling = lingual cortex, precun = precuneus regions.

In Fig. 6 and Table 3, the correlation between |DCS| value and τ PET SUVr is explored. Among the limbic ROIs, the parahippocampal cortex and the amygdala regions showed a statistically significant association between the |DCS| value and τ PET; the association remained statistically significant only in the parahippocampal cortex after correction for multiple comparisons. Of the neocortical ROIs, fusiform gyrus, lingual cortex, and precuneus regions showed a strong association between the |DCS| value and τ PET before multiple comparisons correction, where the association strength survived the multiple comparisons correction for the fusiform gyrus. In all the other cortical ROIs the associations, in general, were negative without statistical significance except for the middle temporal gyrus.

**Fig. 7.**
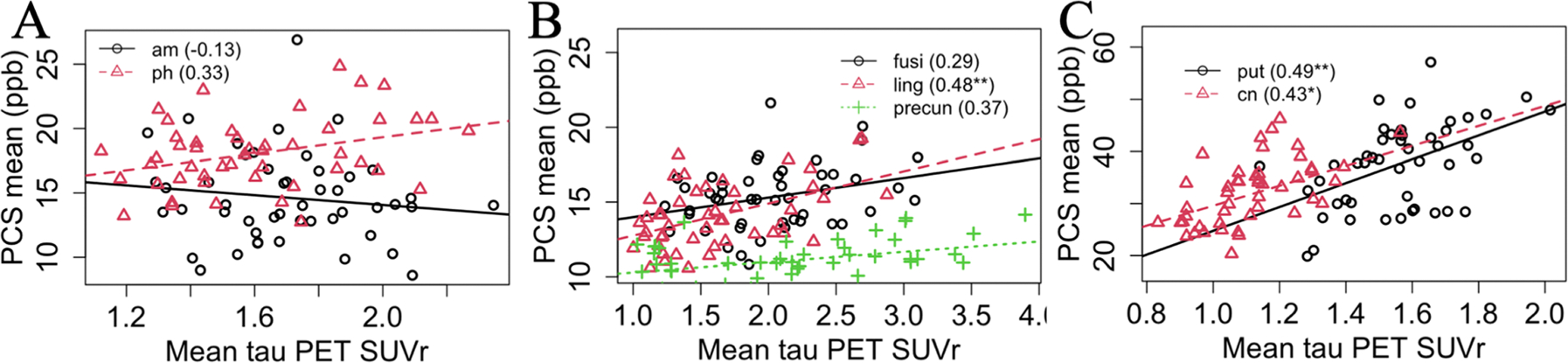
(A) Limbic, (B) neocortical, and (C) subcortical regional association between mean PCS and τ PET for selected ROIs. The number in the parenthesis represents Pearson’s correlation coefficient. Here, *p<0.05, **p<0.01, ***p<0.001, after correction for multiple comparisons using permutation tests. Also, am = amygdala, ph = parahippocampal cortex, fusi = fusiform gyrus, ling = lingual cortex, precun = precuneus, put = putamen, cn = caudate nucleus.

In Fig. 7 and Table 3, the correlations between PCS and τ PET are presented. In Fig. 7(A) the parahippocampal cortex showed a statistically significant association with τ PET before multiple comparisons correction. Amongst the neocortical ROIs, lingual cortex, fusiform gyrus, and precuneus showed statistically significant strong association before multiple comparisons correction as shown in Fig 7(B). After multiple comparisons correction, lingual cortex still showed a significant association. Among the subcortical ROIs, both putamen and caudate nucleus showed statistically significant association before and after multiple comparisons correction. In other ROIs, PCS showed a statistically insignificant positive association with τ PET except for the amygdala and the entorhinal cortex.

The focus of the ROI analysis is on the τ-pathology-affected AD patients. For completeness, we conducted a similar ROI correlation analysis for β-amyloid PET in AD patients; we further repeated the τ and β-amyloid PET analyses for HC participants (supporting materials Tables S1, S2, and S3).

## 4. Discussion

In this work, |DCS| values of aDem participants relative to HC participants, are investigated extensively, as it may provide additional diamagnetic susceptibility-specific information such as protein aggregation and demyelination compared to uncompartmentalized QSM.

### 4.1. Voxel-wise DCS changes in gray matter (GM)

The voxel-wise |DCS| value in several gray matter clusters was higher for aDem patients relative to the HC participants as shown in Fig. 2. One explanation, for the elevated |DCS| value, may be the accumulation of the diamagnetic species. Most proteins contain a high level of paired electrons, hence are diamagnetic. Many efforts have been made to confirm that disease-related aggregations of the β-amyloid plaques and τ tangles cause the tissue susceptibility changes and can be non-invasively revealed by QSM.

Previously, van Bergen et al. (2018) demonstrated that β-amyloid PET SUVr was positively associated with mean QSM in each different cortical region for every subject and Spotorno et al. (2020) showed τ PET SUVr also was positively associated with regional QSM. The positive association between PET SUVr and QSM was explained by the colocalization of iron with the plaques and tangles. However, the missing piece was that the susceptibility sources were not separated, as a result, the diamagnetic component was likely overwhelmed by the paramagnetic susceptibility of iron. Gong et al. (2019) showed that β-amyloid and τ protein are diamagnetic and visible through QSM using phantom experiments. Additionally, in the same study, it is shown that for a human brain specimen with AD, the diamagnetic pattern in QSM aligns with the histological staining for β-amyloid plaques and τ tangles. Zhao et al. (2021) showed that in AD-diagnosed human hippocampal slices, the aggregations of β-amyloid and τ cause diamagnetic changes in QSM. In the current work, |DCS| value of β-amyloid and τ positive AD patients being higher in several regions than that of their β-amyloid and τ negative HC counterparts may allude to the existence of voxels with accumulated plaques and tangles not just iron.

Further investigation reveals that some of the clusters with higher absolute |DCS| values in aDem patients compared to HC participants were either situated in the boundaries of the GM and WM or seemed to appear to have much smaller dimensions compared to the spread of the PET signal intensity as shown in Fig. 2 or both. This mismatch of dimension can be attributed partly to the resolution mismatch between PET and DECOMPOSE-QSM. Besides, it is worth mentioning that the susceptibility is not a direct measurement of the existent β-amyloid or τ protein aggregations, other species such as myelin also contribute to the DCS contrast. Demyelination may result in lower |DCS| as shown in Fig. S3. So, in a region depending on the severity and the order of the arrival of either pathological alteration, such as protein accumulation or demyelination |DCS| may show higher or lower values.

### 4.2. Voxel-wise DCS changes in white matter (WM)

Shown in Fig. 3, regions of both higher and lower |DCS| values are observed for the aDem patients relative to the HC participants. The number of voxels with lower |DCS| value is higher in the dementia group both visually (Fig. 3) and quantitively (Table 2). A lower trend of |DCS| value indicates a decreased amount of diamagnetic species. Previously, Acosta-Cabronero et al. (2013) reported that not many clusters with lower susceptibility in AD compared to controls were found comparing corresponding QSM. It can be argued, that because non-compartmentalized QSM was used in that work and the number of subjects was very small (19 in total), it was difficult to find significant clusters with lower absolute susceptibility in AD patients. Studying a relatively small number of AD (10) patients Au et al. (Au et al., 2021) showed an increase of QSM in a very small structure known as hippocampal fimbria ascribing the increase to iron accumulation. In current work, some clusters were also seen with higher |DCS| in aDem patients in Fig. 3, however, the majority (in the number of voxels) of the clusters showed lower |DCS| compared to that of HC participants. On top of that, Gong et al. (Gong et al., 2023) showed that lower myelin content is associated with rapid cognitive decline.

Previously in a study by Liu et al. (2011) a dramatic reduction of susceptibility in WM was observed in shiverer mice as an indication of the loss of myelin. Meanwhile, diffusion measurements showed intact fiber pathways. Similarly, a decreased diamagnetism (an increase in signed QSM value) in WM was observed by Lee et al. (2012) and Wang et al. (2019) independently through the Cuprizone diet-induced demyelinated mouse model. While the fractional anisotropy (FA) map from diffusion tensor imaging showed the preservation of the axonal structure in the CC, the QSM measured susceptibility was significantly increased (less negative) in CC for the demyelinated mouse model. The QSM value in CC returned to negative during the remyelination process. A study by O’Callaghan et al. (2017) showed that the WM/GM contrast was reduced for the mouse with τ pathology compared to the wild type. Further, the histology staining revealed the white matter atrophy in CC, and the white matter structure thickness is significantly decreased for τ pathology-affected mice. All the evidence suggests that myelin is the major diamagnetic contribution in QSM contrast in the WM. Previously in various studies (Brickman, 2013; Brickman et al., 2014; Capizzano et al., 2004; Hirono et al., 2000), as an indication of abnormal myelination, white matter hyperintensities from FLAIR MRI scans were observed for AD patients in various cases (Brickman, 2013; Brickman et al., 2014; Capizzano et al., 2004; Hirono et al., 2000). The β-amyloid aggregation in cortical GM is hypothesized to participate in a cascade process and cause WM abnormalities in AD patients (Nasrabady et al., 2018; Bouhrara et al., 2018). The multi-layered myelin sheath is crucial for the axon to perform proper functions in the nervous system. The loss of myelination would cause axonal damage and eventually affect the behavior (McKenzie et al., 2014). Although the aggregations of β-amyloid and τ proteins happen in the WM as well (O’Callaghan et al., 2017; Holmes et al., 2016), the PET imaging results suggest that the plaques mostly exist in the GM rather than the WM (Fig. 2). The size of plaques is very small relative to the clinical imaging resolution. Therefore, in WM, the dominant cause of WM susceptibility change is the alteration of myelination. With all these considerations, the observed lower |DCS| value in aDem patients was likely caused by the loss of myelination during AD progression.

### 4.3. Voxel-wise DCS changes in cerebrospinal fluid (CSF)

A lower |DCS| value in the CSF regions was observed for the aDem patients relative to the HC participants. CSF serves as the support fluid of the whole brain volume. Through the blood brain barrier, CSF carries biomolecules such as proteins and peptides that can directly indicate the active inflammation and disease pathologies of the brain. There are many studies focusing on τ and β-amyloid in the CSF as biomarkers for AD (Blennow et al., 2010; Perrin et al., 2009; Blennow and Hampel, Oct. 2003). β-amyloid-42 is the dominant component of the plaques seen in AD (Roher et al., 1993), the decreased CSF β-amyloid-42 or the decrease of CSF β-amyloid-42/β-amyloid-40 ratio has been reported to be superior in identifying patients with AD (Hansson et al., 2019). Thal et al. (2006) showed that the β-amyloid-40 stays unchanged for AD cases. Additionally, the increase of CSF phosphorylated-τ/β-amyloid-42 ratio is shown to be an accurate biomarker for detecting AD dementia (Bouwman et al., 2022). Therefore, the decrease of β-amyloid-42 in CSF may be one of the important contributing factors behind the reduction of the β-amyloid-42/β-amyloid-40 ratio. Although |DCS| maps from DECOMPOSE-QSM cannot give the susceptibility species composition, a decreased trend of the |DCS| value would suggest a reduced total protein content and seems to agree with the report of the decreased level of β-amyloid-42 or the decreased ratio of β-amyloid-42/ β-amyloid-40. However, this finding needs to be verified ideally with CSF samples (Choi et al., 2022).

Alternatively, a previous AD study by Choi et al. (2022) looked into the choroid plexus (CP), a structure that produces cerebrospinal fluid. It showed that the volume of CP is higher for the AD group compared to the cognitively impaired group while the susceptibility of CP did not show significant change. The changes in the volume of CP could contribute to the observation of |DCS| changes as well, however, the effect is unclear. It is also worth mentioning that the clusters with lower |DCS| values in AD patients compared to HC participants appear to align with the periphery of the ventricles. Impaired barriers caused by ependymal cells and vessels may also play a role in the change of DCS in the CSF region (Nelles and Hazrati, 2022). The bulk magnetic susceptibility in CSF is supposed to be close to zero and its long T2* makes susceptibility estimation less reliable. Moreover, partial volume effects, artifacts, and CSF widening can confound the susceptibility metrics. To elaborate, as shown in Fig. 1 the ventricular area is increased in aDem patients. As a result, CSF voxels in aDem brains may overlap with some WM voxels in HC brains. CSF have lower |DCS| compared to WM, which, may explain the reduced |DCS| in aDem brains in the boundary regions of CSF and WM. Therefore, CSF enlargement explained the reduction of |DCS| partially. However, the clusters of differences extended beyond the CSF and WM tissue boundary. Hence, direct comparison using CSF samples would be necessary to verify and explain our findings.

### 4.4. Correlation analysis with τ pathology for cortex and deep gray matter ROIs

τ pathology has been known to be more associated with cognitive decline in Alzheimer’s disease compared to that of β-amyloid (Pontecorvo et al., 2017; Young et al., 2020). Hence, the ROIs that are known to be affected by τ pathology, have been selected in the study.

In Fig. 6, in several limbic and cortical ROIs, mean τ PET was associated with mean |DCS| value moderately (Pearson’s correlation coefficient > 0.3) or strongly (Pearson’s correlation coefficient > 0.5). This relationship may seem contrary to what was observed in Fig. 2, where a higher |DCS| value was seen in several GM voxel clusters. However, it should be noted that Fig. 2 is showing the voxel-wise comparison between aDem patients and controls. On the other hand, Fig. 6 is showing ROI correlation for dementia patients between the ROI mean measures. It is possible that in the ROI level after averaging, only the strongest of the several competing effects dominate mean |DCS|. The competing effects may be protein accumulations (Gong et al., 2019), cell death (Nirzhor et al., 2018), atrophy (Nasrabady et al., 2018), and demyelination (Bouhrara et al., 2018). Protein accumulation may increase |DCS| values and demyelination may reduce the |DCS| values. Cell death and atrophy may affect |DCS| values in a complicated fashion that is yet to be explored. The negative direction of association suggests that the demyelination is potentially the leading factor contributing to smaller absolute diamagnetic susceptibility with a larger τ PET signal. Previously, Irimia et al. (2021) reported a reduction in the T1-w/T2-w ratio (a marker of myelin content) in some frontal and central regions while Pelkmans et al. (2019) reported an increase of the same in some ROIs including cingulate gyrus, and precuneus. So, conflicting reports have been made on whether demyelination in GM happens measurably using MRI-derived contrast; DCS should be included among one of those contrasts for further investigation.

PCS and τ PET association was also investigated within the ROI-based analysis. In the subcortical ROIs PCS was strongly associated with τ PET. However, the range of τ PET SUVr variation is small in these ROIs. In the subcortical ROIs FTP PET results in off-target binding related to potential inflammation; so subsequent iron accumulation may be the origin of the positive association (Cogswell et al., 2021). In other cortical ROIs τ PET showed a positive association with PCS. This may happen because of the colocalization of iron with τ tangles (Spotorno et al., 2020). These observations matching previous publications may increase the confidence in the conclusions acquired from the ROI-based DCS analysis.

Turning to association between amyloid PET vs PCS and |DCS| as presented in Table S2 none of the correlation coefficients survive statistical significance test for the aDem patients. Acknowledging that the ROIs chosen for the current analysis are primarily affected by τ pathology this is not surprising. Although previous studies (van Bergen et al., 2018; Ayton et al., 2017; Chen et al., 2021; Van Bergen et al., 2016) demonstrated association between amyloid PET and QSM the susceptibility values were not compartmentalized. The study by Ayton et al. (Ayton et al., 2017) contained only 19 AD patients and showed association between QSM and PET in larger brain regions such as frontal, temporal, and occipital lobes. Van Bergen et al. (2016) studied patients with MCI and showed difference using QSM. In other studies, van Bergen et al. (2018) and Chen et al. (2021) reported similar association using QSM in cognitively older adults. The varying choice of regions of interest and use of different metric may explain difference between current work and previous research.

### 4.5. Limitations and future directions

There are numerous studies on using QSM as a potential biomarker for neurodegenerative diseases. The purpose of this work was to investigate whether the two derived contrasts, namely PCS and DCS, from DECOMPOSE-QSM, can provide additional information. To gain a comprehensive view of how much DECOMPOSE could be beneficial to understand AD progression, many other contrasts and clinical diagnosis data need to be applied in parallel. Histological verification was out of scope in the current study, which would be needed in the future to verify the usefulness of DCS. This verification can ensure whether the gray matter susceptibility changes detected in the current work represent demyelination. Due to the nature of the retrospective analysis, the MEGRE data of the participants used in the current work only contain five echoes which is the minimum number of echoes for DECOMPOSE to perform in theory, and more echoes could benefit better susceptibility compartmentalization. The DECOMPOSE-QSM model does not address the composition of species within the diamagnetic or paramagnetic regime. We are unable to differentiate which protein or peptide specifically contributes to the DCS contrast. Longitudinal study rather than cross-sectional analysis may provide more meaningful insight into the relationship between pathology progression and susceptibility measures as well. Statistical tests have been used to assess the relationship between pathological markers and susceptibility. These tests are limited by the number of participants and the noise level of data. A conservative family-wise correction was applied for all p-value-based statistics to increase their reliability. In addition to the p-values, the magnitude of the correlation coefficient was taken as a convincing measure of association.

## 5. Conclusion

The diamagnetic compartment DCS from DECOMPOSE-QSM is tested as a marker for the presence of pathology (β-amyloid and τ). It appears DCS is affected in an opposing manner by two important pathological changes, such as protein accumulation and demyelination. In the voxel-wise analysis of the WM, a lower absolute value of DCS pointed to white matter integrity loss. Voxel-wise analysis results of the |DCS| value in the CSF region may be an indication of the decrease of the β-amyloid-42/ β-amyloid-40 ratio in AD. In short, diamagnetic susceptibility may help track several pathological alterations in neurodegeneration. The distinctive way of measuring compartmentalized magnetic susceptibility will allow the investigation of pathological changes happening in all tissue types such as GM, WM, and CSF of the brain.

## Supplementary Material

supplementary

Supplementary material associated with this article can be found, in the online version, at doi:10.1016/j.neuroimage.2023.120357.

## Figures and Tables

**Table 1:** Demographic details of all participant data.

Variable	aDem Patients (n=49)	CU HC participants (n=71)

Male/female	24/24 (50%/50%)	42/29 (59%/41%)
Age	67 (60, 76)	65 (52, 71)
STMS	22 (18, 28)	37 (36, 38)
Education	16 (13.75, 16)	16 (14, 16.5)

Values are displayed as median (1^st^ quartile, 3^rd^ quartile). STMS: Short Test of Mental State.

**Table 2:** Ratio of voxels with statistically significant (p<0.05) higher PCS or |DCS| in aDEM patients compared to the HC participants as defined in Eqs. (1) & (2).

Segmentation/metric	*m*_PCS_	*m*_|DCS|_

GM	4.39	0.64
WM	6.52	0.34
CSF	4.78	0.19

**Table 3:** Regional correlation between τ PET and PCS or |DCS| for amyloid and τ positive aDEM patients.

ROI	PCS vs τ PET			|DCS| vs τ PET		
		
	Pearson’s r	p-value	corrected p-value	Pearson’s r	p-value	corrected p-value

Entorhinal cortex	−0.05	0.73	1.00	−0.25	0.08	0.64
Parahippocampal cortex	0.33	0.02*	0.23	−0.57	<0.001***	<0.001***
Inferior temporal cortex	0.21	0.15	0.84	−0.11	0.45	0.999
Middle temporal cortex	0.15	0.32	0.99	−0.01	0.27	1.00
Superior temporal cortex	0.21	0.15	0.83	−0.22	0.14	0.82
Fusiform gyrus	0.29	0.05*	0.44	−0.48	<0.001***	0.007**
Lingual cortex	0.48	<0.001***	0.007**	−0.33	0.02*	0.22
Precuneus	0.37	0.009**	0.1	−0.34	0.02*	0.21
Posterior cingulate cortex	0.11	0.44	0.999	−0.08	0.6	1.00
Amygdala	−0.13	0.37	0.99	−0.35	0.01*	0.14
Putamen	0.49	<0.001***	0.006**	0.08	0.57	1.00
Caudate nucleus	0.43	0.002**	0.03*	0.16	0.42	0.98
Globus pallidus	0.27	0.07	0.55	0.28	0.05	0.47

## Data Availability

MRI, PET, and other data from the Mayo Clinic Alzheimer’s Disease Resrearch Center (MCADRC) are available to qualified academic and industry researchers by request to the MCADRC Executive Committee

## Abbreviations

MEGRE: Multi-Echo Gradient Recalled Echo
QSM: Quantitative Susceptibility Mapping
DECOMPOSE: DiamagnEtic COMponent and Paramagnetic cOmponent SEparation
DCS: Diamagnetic Component Susceptibility
PCS: Paramagnetic Component Susceptibility
aDem: amnestic Dementia
