# Supplementary material for: The diamagnetic component map from quantitative susceptibility mapping (QSM) source separation reveals pathological alteration in Alzheimer’s disease-driven neurodegeneration
Source: Neuroimage. Author manuscript; Available in PMC 2026 Mar 25. (PMC13014792; doi:10.1016/j.neuroimage.2023.120357)
Supplement: supplementary [file NIHMS2144581-supplement-supplementary.docx]

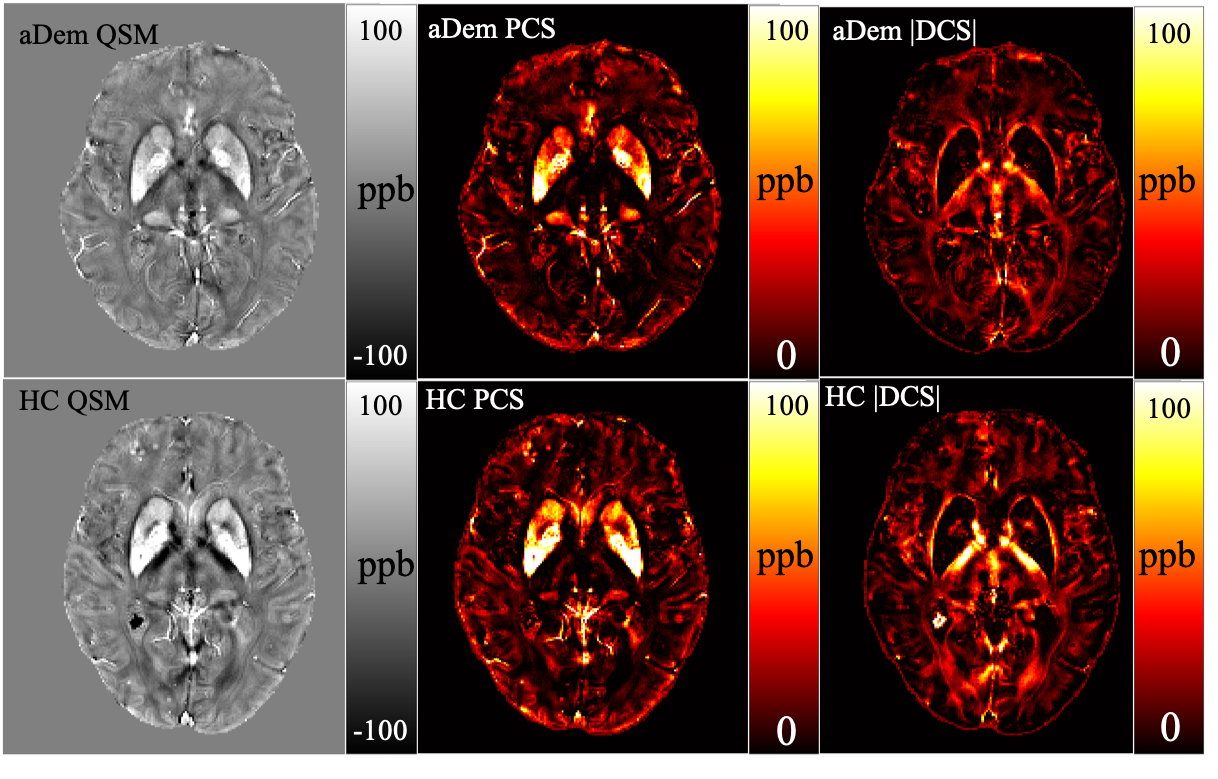


Fig. S1: Different susceptibility images collected and used in the analysis. Images in the top panel come from an 89-year-old aDem patient and the bottom panel come from a 73-year-old healthy control subject in their corresponding native spaces. ppb: parts per billion, 10^-9^.


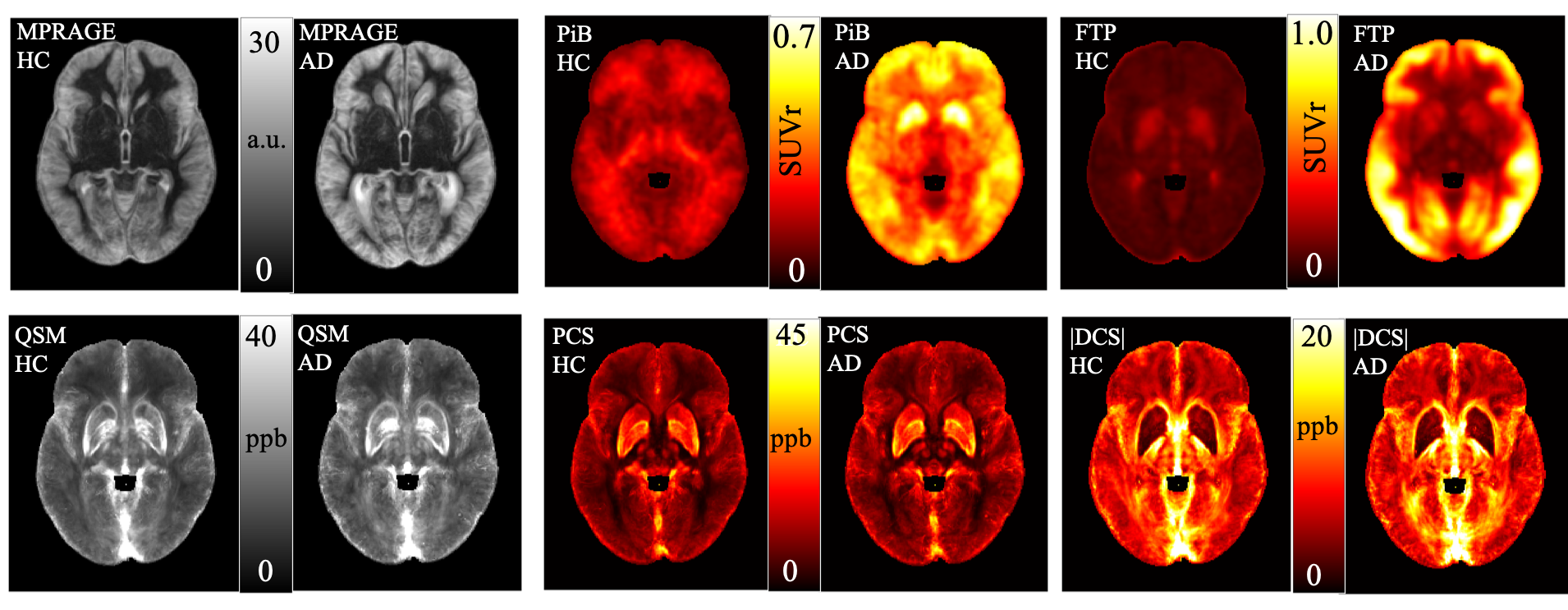
Fig. S2: Standard deviation of all the modalities of images for both aDem and HC groups. The prominent difference can be seen in basal ganglia for QSM and PCS maps whereas cortical WM in frontal lobe is seen in |DCS| maps.


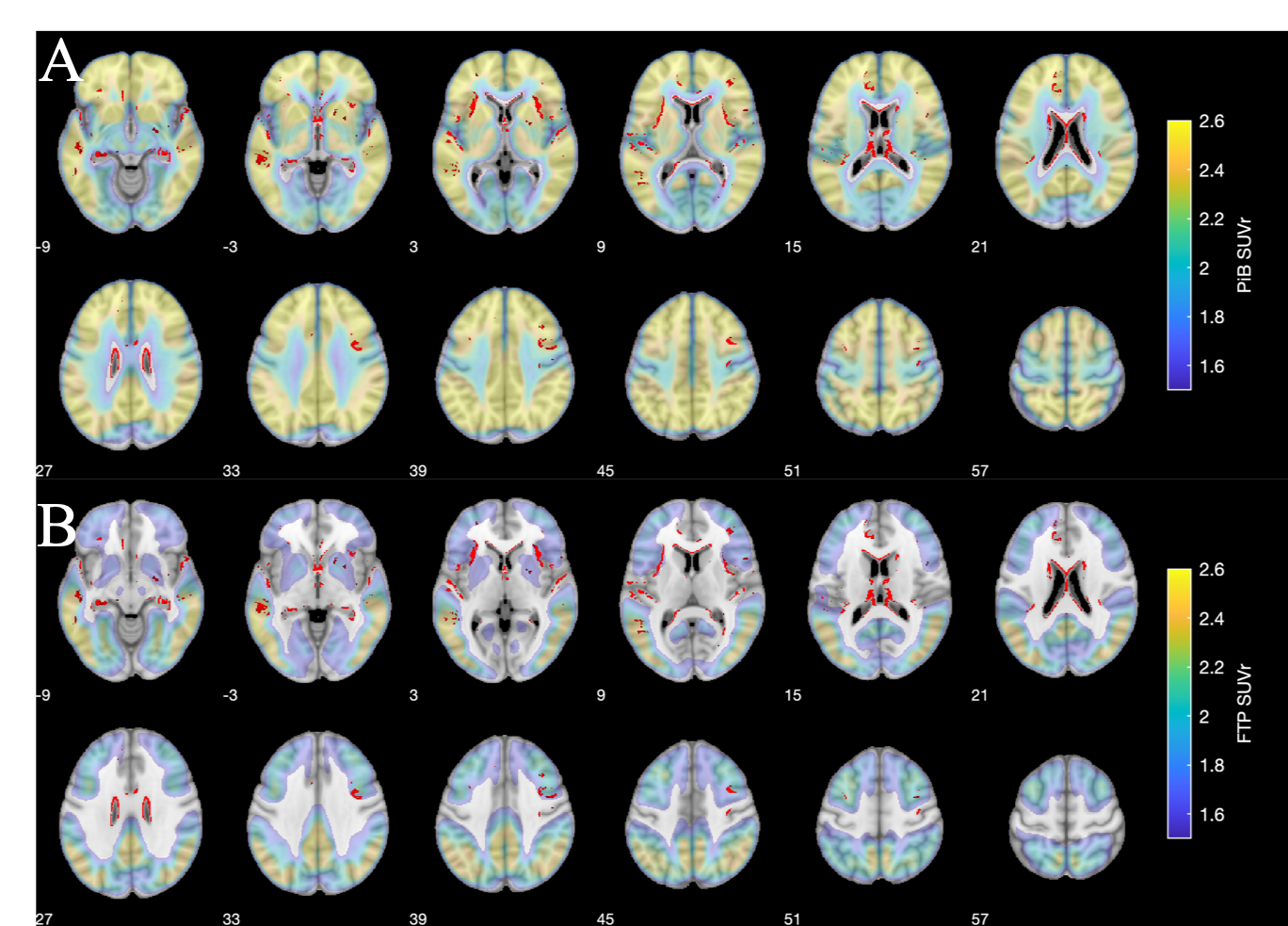


Fig. S3: Regions of statistically significant (p<0.05) lower |DCS| (red) in the GM of the aDem patients vs CU HC overlaid on top of the average β-amyloid PET (A, top two rows) and average 𝜏 PET (B, bottom two rows) in MNI space. A few of the clusters overlap with the highest intensity PET signal, which may correspond to the demyelinated region discussed in the corresponding article. Some of the clusters reside in tissue segmentation boundaries, particularly in the subcortical and thalamic regions may not be directly associated with the PET signal. However, these differences still can be ascribed to pathological differences between aDem and HC groups.


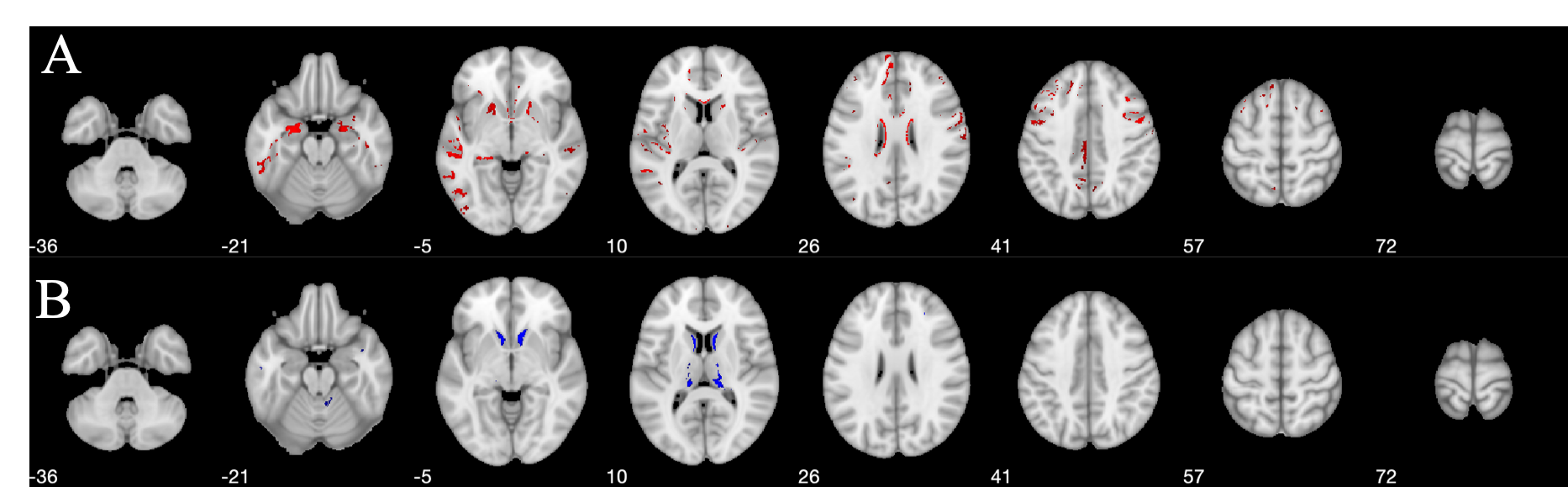


Fig. S4: Regions of statistically significant (p<0.05) (A) higher (red) and (B) lower (blue) PCS in the GM of amnestic dementia patients relative to the healthy control participants in MNI space. Most clusters with higher PCS in aDem group reside in the cortical regions because of the iron accumulation associated with β-amyloid or 𝜏 pathology. A few clusters with higher PCS also reside in the Putamen region which has been shown to have higher QSM in AD patients in a previous report [1]. A few clusters with lower PCS in aDem group reside in the subcortical caudate nucleus and thalamic regions. These regions have not shown a significant difference in some previous reports utilizing QSM [1], [2] whereas in others caudate nucleus showed higher QSM in AD patients compared to healthy controls [3], [4].


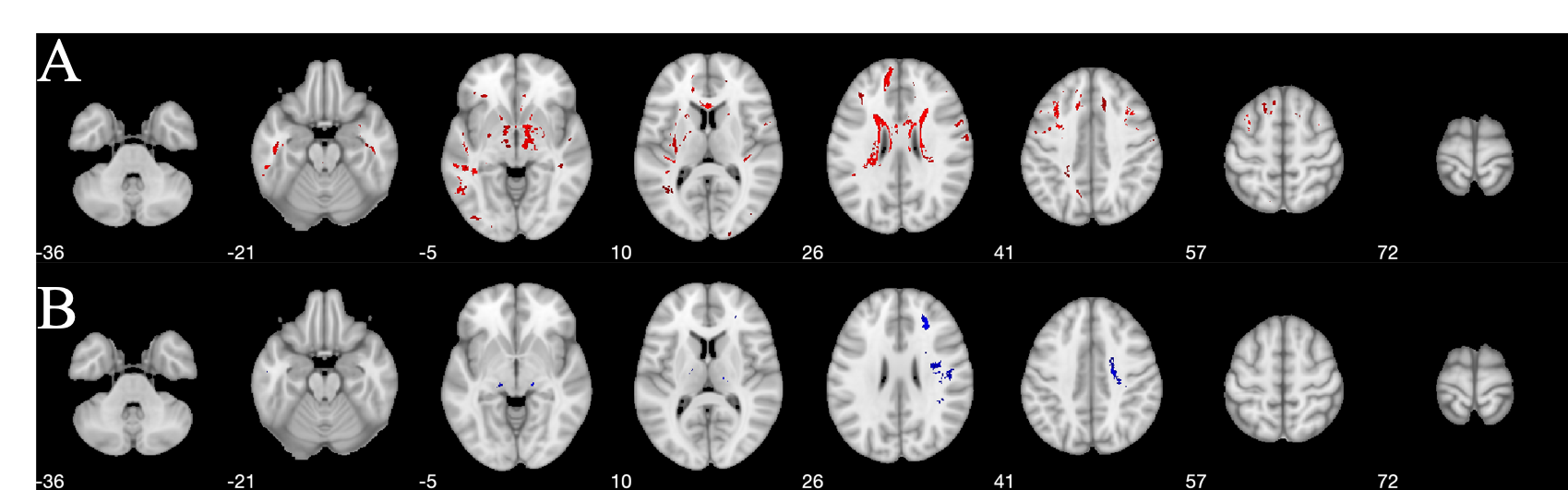


Fig. S5: Regions of statistically significant (p<0.05) (A) higher (red) and (B) lower (blue) PCS in the WM of amnestic dementia patients relative to the healthy controls in MNI space.


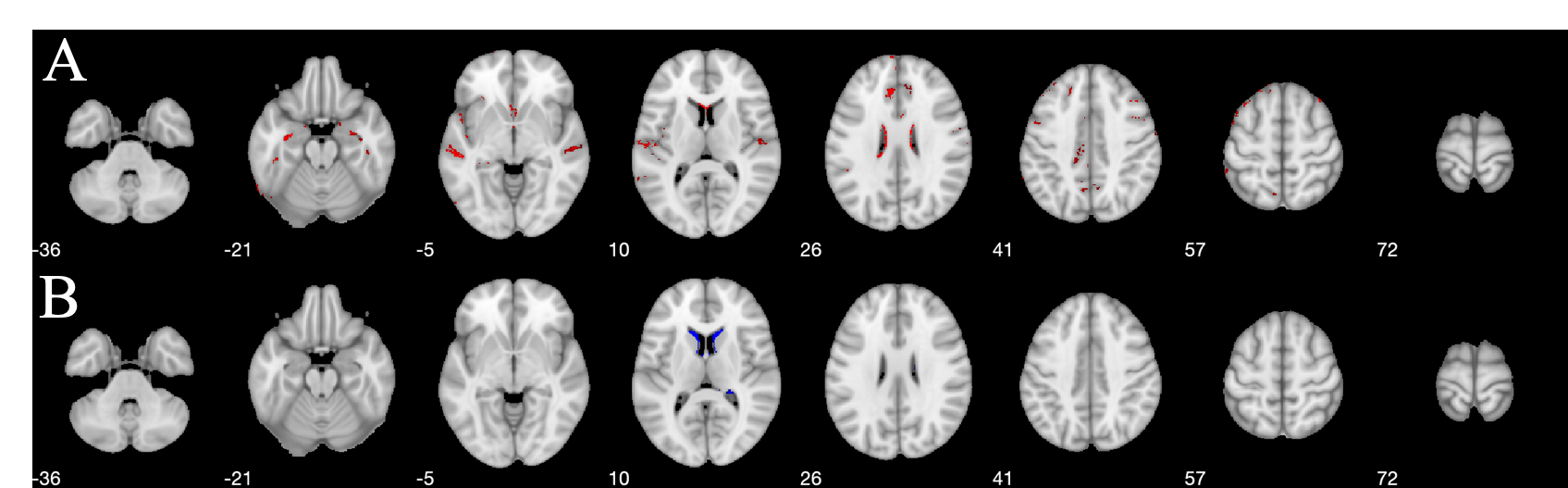


Fig. S6: Regions of statistically significant (p<0.05) (A) higher (red) and (B) lower (blue) magnitude PCS in the CSF of aDem patients compared to the HC participants in MNI space. There are more clusters in higher PCS images for aDem group compared to the lower PCS image.


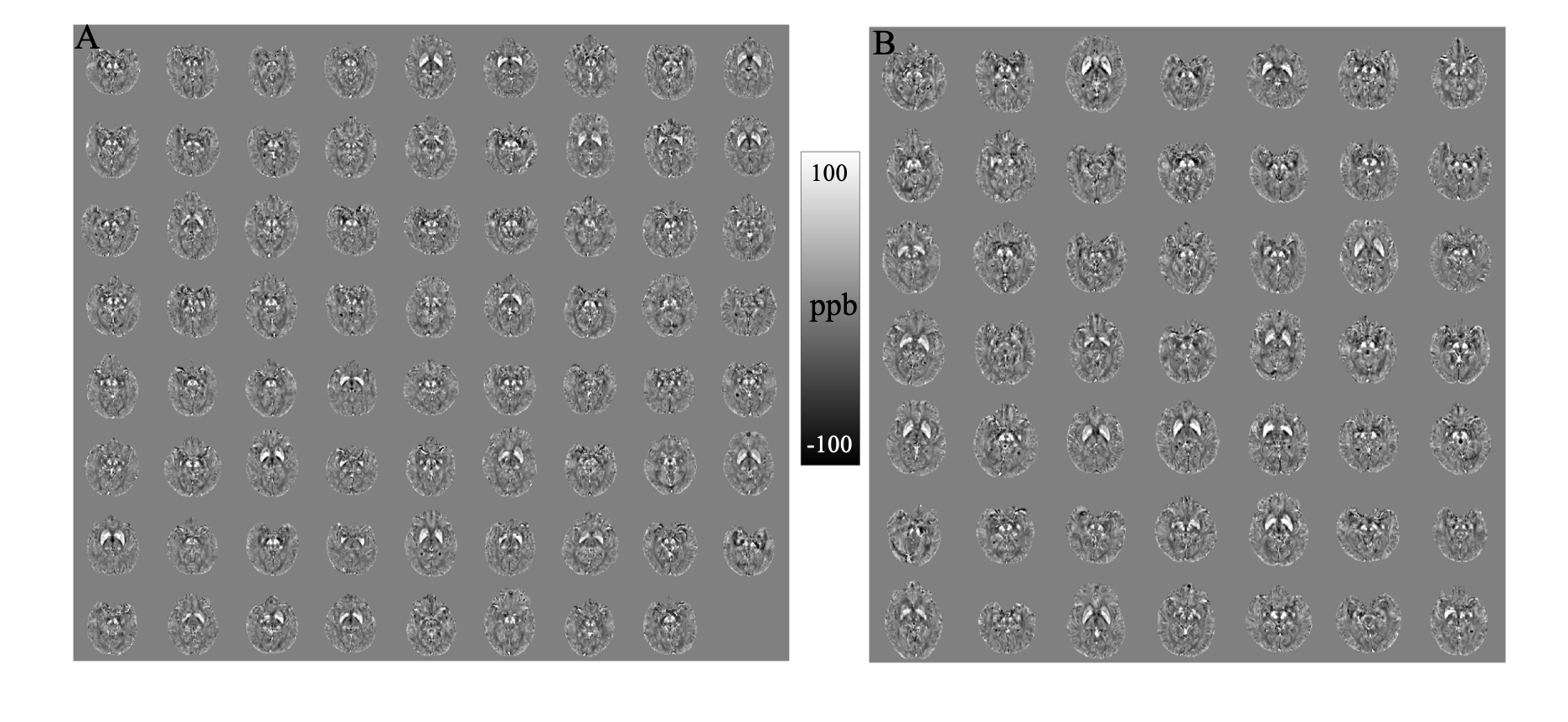


Fig. S7: Montage of one slice of QSM images of (A) HC participants, and (B) AD patients used in the current study. Slice locations are different for different subjects because slices were collected from native spaces of the images which are not aligned with each other.

| ROI | PCS vs 𝜏 PET | | | \|DCS\| vs 𝜏 PET | | |
| --- | --- | --- | --- | --- | --- | --- |
|  | Pearson’s r | p-value | corrected p-value | Pearson’s r | p-value | corrected p-value |
| Entorhinal cortex | 0.12 | 0.33 | 0.99 | -0.001 | 0.99 | 1.00 |
| Parahippocampal cortex | 0.18 | 0.14 | 0.82 | -0.19 | 0.12 | 0.7 |
| Inferior temporal cortex | 0.14 | 0.23 | 0.95 | 0.25 | 0.04* | 0.33 |
| Middle temporal cortex | 0.006 | 0.96 | 1.00 | 0.24 | 0.04* | 0.34 |
| Superior temporal cortex | 0.54 | <0.001*** | 0.001** | 0.26 | 0.03* | 0.25 |
| Fusiform gyrus | 0.26 | 0.03* | 0.3 | 0.03 | 0.86 | 1.00 |
| Lingual cortex | 0.07 | 0.57 | 0.999 | -0.07 | 0.59 | 1.00 |
| Precuneus | 0.04 | 0.74 | 1.00 | -0.05 | 0.67 | 1.00 |
| Posterior cingulate cortex | 0.17 | 0.16 | 0.87 | -0.11 | 0.35 | 0.99 |
| Amygdala | 0.06 | 0.62 | 1.00 | -0.03 | 0.78 | 1.00 |
| Putamen | 0.51 | <0.001*** | 0.004** | 0.31 | 0.007** | 0.08 |
| Caudate nucleus | 0.09 | 0.47 | 0.999 | 0.24 | 0.04* | 0.37 |
| Globus pallidus | 0.22 | 0.07 | 0.55 | 0.05 | 0.66 | 1.00 |

Table S1: Regional correlation between 𝜏 PET and PCS or DCS for amyloid and 𝜏 negative HC participants

| ROI | PCS vs amyloid PET | | | \|DCS\| vs amyloid PET | | |
| --- | --- | --- | --- | --- | --- | --- |
|  | Pearson’s r | p-value | corrected p-value | Pearson’s r | p-value | corrected p-value |
| Entorhinal cortex | 0.02 | 0.91 | 1.00 | -0.27 | 0.06 | 0.49 |
| Parahippocampal cortex | 0.1 | 0.49 | 0.999 | -0.06 | 0.67 | 1.00 |
| Inferior temporal cortex | 0.07 | 0.64 | 1.00 | 0.12 | 0.43 | 0.99 |
| Middle temporal cortex | 0.05 | 0.74 | 1.00 | 0.07 | 0.64 | 1.00 |
| Superior temporal cortex | 0.11 | 0.46 | 0.999 | 0.04 | 0.78 | 1.00 |
| Fusiform gyrus | 0.07 | 0.63 | 1.00 | -0.006 | 0.97 | 1.00 |
| Lingual cortex | 0.12 | 0.41 | 0.998 | -0.14 | 0.33 | 0.99 |
| Precuneus | -0.01 | 0.94 | 1.00 | 0.15 | 0.3 | 0.98 |
| Posterior cingulate cortex | 0.05 | 0.75 | 1.00 | 0.03 | 0.84 | 1.00 |
| Amygdala | -0.01 | 0.93 | 1.00 | 0.07 | 0.66 | 1.00 |
| Putamen | 0.17 | 0.22 | 0.95 | 0.03 | 0.85 | 1.00 |
| Caudate nucleus | 0.05 | 0.73 | 1.00 | 0.27 | 0.07 | 0.52 |
| Globus pallidus | 0.08 | 0.58 | 1.00 | 0.01 | 0.97 | 1.00 |

Table S2: Regional correlation between amyloid PET and PCS or DCS for amyloid and 𝜏 positive AD patients

| ROI | PCS vs amyloid PET | | | \|DCS\| vs amyloid PET | | |
| --- | --- | --- | --- | --- | --- | --- |
|  | Pearson’s r | p-value | corrected p-value | Pearson’s r | p-value | corrected p-value |
| Entorhinal cortex | 0.26 | 0.03* | 0.29 | 0.17 | 0.16 | 0.8 |
| Parahippocampal cortex | 0.21 | 0.08 | 0.6 | 0.35 | 0.002** | 0.03* |
| Inferior temporal cortex | 0.21 | 0.07 | 0.58 | 0.38 | 0.0012** | 0.012* |
| Middle temporal cortex | 0.12 | 0.33 | 0.98 | 0.47 | <0.001*** | <0.001*** |
| Superior temporal cortex | 0.27 | 0.02 | 0.23 | 0.37 | 0.0011** | 0.01* |
| Fusiform gyrus | 0.12 | 0.33 | 0.99 | 0.39 | <0.001*** | 0.008** |
| Lingual cortex | 0.01 | 0.93 | 1.00 | 0.17 | 0.16 | 0.81 |
| Precuneus | -0.14 | 0.25 | 0.96 | 0.28 | 0.02* | 0.17 |
| Posterior cingulate cortex | -0.25 | 0.03* | 0.31 | 0.4 | <0.001*** | 0.005** |
| Amygdala | 0.18 | 0.13 | 0.79 | 0.26 | 0.03* | 0.27 |
| Putamen | 0.41 | <0.001*** | 0.005** | 0.3 | 0.012* | 0.13 |
| Caudate nucleus | 0.05 | 0.68 | 1.00 | 0.2 | 0.1 | 0.64 |
| Globus pallidus | 0.05 | 0.69 | 1.00 | 0.21 | 0.07 | 0.53 |

Table S3: Regional correlation between amyloid PET and PCS or DCS for amyloid and 𝜏 negative HC participants

References

[1] S. Tiepolt *et al.*, “Quantitative susceptibility mapping in β-Amyloid PET-stratified patients with dementia and healthy controls – A hybrid PET/MRI study,” *Eur J Radiol*, vol. 131, no. October 2019, p. 109243, 2020, doi: 10.1016/j.ejrad.2020.109243.

[2] H. G. Kim *et al.*, “Quantitative susceptibility mapping to evaluate the early stage of Alzheimer’s disease,” *Neuroimage Clin*, vol. 16, no. March, pp. 429–438, 2017, doi: 10.1016/j.nicl.2017.08.019.

[3] L. Du *et al.*, “Increased Iron Deposition on Brain Quantitative Susceptibility Mapping Correlates with Decreased Cognitive Function in Alzheimer’s Disease,” *ACS Chem Neurosci*, vol. 9, no. 7, pp. 1849–1857, Jul. 2018, doi: 10.1021/acschemneuro.8b00194.

[4] Y. Moon, S.-H. Han, and W.-J. Moon, “Patterns of Brain Iron Accumulation in Vascular Dementia and Alzheimer’s Dementia Using Quantitative Susceptibility Mapping Imaging,” *Journal of Alzheimer’s Disease*, vol. 51, pp. 737–745, 2016, doi: 10.3233/JAD-151037.
